# Radiotherapy or surgery for spine metastases? A population-based study of 903 patients in the south-eastern region of Norway

**DOI:** 10.3109/17453674.2011.566142

**Published:** 2011-07-08

**Authors:** Olga Zaikova, Sophie D Fosså, Øyvind S Bruland, Karl-Erik Giercksky, Berit Sandstad, Sigmund Skjeldal

**Affiliations:** ^1^Department of Orthopaedics; ^2^Division of Surgery and Cancer Medicine, Norwegian Radium Hospital, Oslo University Hospital; ^3^Institute of Clinical Medicine, Faculty of Medicine, University of Oslo; ^4^Clinical Trials Unit, Norwegian Radium Hospital, Oslo University Hospital, Oslo, Norway

## Abstract

**Background and purpose:**

Radiotherapy (RT) remains the cornerstone of management of spine metastases (SM), even though surgery is a well-established treatment for selected patients. We compared the use of RT and surgery in a population-based cohort of patients with SM, investigated pre-treatment factors that were associated with use of these treatment modalities, and examined survival.

**Patients and methods:**

903 patients in the south-eastern Norway who were admitted for RT or surgery for SM for the first time during an 18-month period in 2007–2008 were identified and their medical records were reviewed.

**Results:**

The primary treatment was surgery in 58 patients and RT in 845 patients, including 704 multiple-fraction (MF) and 141 single-fraction (SF) RT schedules. 11 of 607 patients without motor impairment (2%) and 47 of 274 patients with motor impairment (17%) underwent primary operations. 11 of 58 operated patients and 244 of 845 irradiated patients died within 2 months after the start of treatment. 26% of those who received multiple-fraction RT or surgery died within 2 months.

**Interpretation:**

Motor impairment was the main indication for surgery. Better identification of patients with short survival is needed to avoid time-consuming treatment (major surgery and long-term RT).

External-beam RT has been the standard treatment for spine metastases for decades. The effect of surgical treatment as opposed to RT has been widely discussed, but there have been few publications comparing these two treatment modalities (Finkelstein et al. 2003, Bauer 2005, Klimo et al. 2005, Loblaw et al. 2005, Prasad and Schiff 2005, Abrahm et al. 2008). Laminectomy without stabilization appears to give no benefit compared to RT alone (Young et al. 1980). A prospective randomized study comparing surgical decompression and stabilization followed by RT with RT alone in patients with spine metastases (SM) and neurological compromise showed that those who underwent surgery regained the ability to walk more often and maintained the function longer than patients treated with RT alone (Patchell et al. 2005). To our knowledge, no reports comparing surgery and RT with pain relief as outcome have been published. In a Canadian population-based study of malignant spinal cord compression (Loblaw et al. 2003), one fifth of those who underwent RT or surgery were operated initially, but the pretreatment motor status and the indications for surgery were not reported.

Life expectancy for most patients with SM is usually limited to a few months, but varies from weeks to years (van der Linden et al. 2005, Rades et al. 2006, Conway et al. 2007). Although pain relief and preservation of function are the primary aims of the treatment, the choice of treatment modality should also be tailored to the expected survival. Major surgical treatment for SM is generally not indicated if the patient is expected to survive less than 3 months (White et al. 2008). For these patients, the single-fraction (SF) RT schedule is preferable as, according to several studies, SF and multiple-fraction (MF) RT provide equal palliation for painful bone metastases (van der Linden et al. 2004, Hartsell et al. 2005, Kaasa et al. 2006, Chow et al. 2007, Bruland et al. 2009, Sande et al. 2009). A decision-making process on the choice of treatment options should include benefits and complications of each treatment modality based on the results of valid clinical studies that are not biased by selection (Abrahm et al. 2008). Studies on surgery for SM have reported complication rates of 20–30% (Finkelstein et al. 2003, Jansson and Bauer 2006), while serious complications related to palliative RT have not been reported.

In this retrospective study, we examined the recent use of RT and surgery for SM in a population-based cohort of patients in the south-eastern region of Norway and identified pretreatment factors that were associated with the use of either RT or surgery. Furthermore, we calculated the median survival after treatment for SM and analyzed the pretreatment factors that might affect survival.

## Patients and methods

This retrospective study was carried out in the south-eastern region of Norway with a population of 2.6 million inhabitants. All patients with first-time event of either RT or surgery for SM in the cervical, thoracic, or lumbar spine during the time period from February 1, 2007 through July 31, 2008 were identified and included. Exclusion criteria were extraskeletal lesions without affecting osseous components of the vertebrae, intrathecal lesions, and age below 18 years. During the study period, RT was available in 4 hospitals in the region. Surgical treatment of patients with SM was available in 2 hospitals. Although both RT and surgery were available only in 2 of 5 hospitals, surgery alone in 1 hospital, and RT alone in 2 hospitals, a multidisciplinary approach was available for all patients by internet transfer of MRI and discussion between surgical and radiotherapy teams in different hospitals.

For identification of the irradiated patients, the medical records of all patients with radiation target volume in spine were reviewed and checked for eligibility. For operated patients, the surgical departments' procedure lists were checked and the medical records of those who underwent spine surgery for malignancy were reviewed. Only the first event of each treatment modality was recorded for each patient. For patients with more than one cancer diagnosis, only the malignancy with dissemination to the spine was recorded. The treatment modality used initially was defined as the primary treatment.

903 patients were included in this study. We also identified, but excluded, 7 patients who received vertebroplasty as the primary treatment and 98 patients who had at least 1 admission for RT, vertebroplasty, or surgery for SM before the study period. From the medical records we retrieved information on age, sex, dwelling place, type of primary cancer, date of primary cancer diagnosis and diagnosis of SM, ambulatory and neurological status at admission, and the actual treatment modality. We also retrieved routine radiological reports, concentrating on the multiplicity of the affected vertebrae. Frankel grade, which reflects the degree of motor impairment (Frankel et al. 1969), was reconstructed (based on review of the medical records) as follows: A—no motor or sensory function, B—preserved sensation only, no motor function, C—non-ambulatory, wheel-chair bound but some motor function, D—ambulatory, but with neurological symptoms, and E—normal neurological function. Patients with Frankel grades A, B, and C were considered to be non-ambulatory. 23 patients were described as walking with aid, but it was impossible to distinguish between pain and neurological compromise as the principal cause of the impaired ambulatory status. These patients were allocated to Frankel grade D.

RT was applied as high-voltage irradiation with linear accelerators. The target volume included the entire affected vertebrae, the processus transversi and the soft tissue component of the lesion as imaged by CT or MRI. The adjoining proximal and distal vertebrae were included in the treatment volume.

Survival status on April 24, 2010 was provided by the Nowegian National Registry.

### Statistics

The association between categorical variables was evaluated by chi-squared tests (Pearson's and Fisher's exact tests as appropriate). A binary logistic regression model for multiple analysis was used to analyze the significance of pretreatment factors for the choice of primary treatment. This statistical method can overestimate the prevalence ratio, the presented odds ratios, and their confidence intervals, which must therefore interpreted with caution. Kaplan-Meier plots and log-rank tests were used for univariate overall survival analysis. The observation time was from the start of RT or surgery to death, or for a minimum of 19 months. All p-values reported are based on 2-sided tests and a p-value of < 0.05 was considered statistically significant. We used SPSS software for Windows version 16.0.

### Ethics

The study was approved by the Regional Committee for Medical Research Ethics in Southern Norway.

## Results

The median age of the 903 patients was 69 (19–94) years. 542 patients (60%) were men, 260 (29%) had prostate cancer, 179 (20%) had lung cancer, 152 (17%) had breast cancer, 79 had myeloma or lymphoma, 40 patients had kidney cancer, and 193 patients had other cancer diagnoses. 142 (16%) had a solitary metastasis in the spine; the others had multiple spine metastases. 12 of 248 patients with prostate cancer (5%), 21 of 131 patients with breast cancer (14%), 28 of 151 patients with lung cancer (16%), 23 of 56 patients with myeloma or lymphoma, 8 of 23 patients with melanoma, and 11 of 29 patients with kidney cancer had a solitary spine metastasis. In 351 patients (39%), the metastatic lesions in the spine were diagnosed at the time of primary cancer diagnosis, 510 (56%) had no spine metastases at the time of primary cancer diagnosis, and for 42 patients the status was unknown. 845 patients received RT and 58 patients received surgery as primary treatment. The median time from the cancer diagnosis to the first event of RT or surgery was 16 (0–390) months. For the RT group, the median time was 17 (0–390) months while for the surgery group the median time was 0.4 (0–329) months (p < 0.001).

At the start of treatement, 98 patients were non-ambulating—including 9 patients belonging to Frankel group A, 5 patients belonging to Frankel group B, and 84 patients belonging to Frankel group C. 176 patients could walk despite a minor motor deficit (Frankel D) and 607 patients had no motor impairment (Frankel E). The neurological status could not be recorded for 22 patients. Motor impairment was related to the primary cancer diagnosis (Table 1).

**Table 1.** Motor impairment **^a^** at the start of treatment according to the primary cancer diagnosis (p < 0.001) **^b^**

Primary cancer diagnosis	Normal motor status (Frankel E)	Motor impairment	
		Ambulation with minor motor deficit (Frankel D)	No ambulation (Frankel A–C)
Breast	124	18	6
Prostate	170	55	30
Lung	117	37	16
Kidney	30	3	7
Myeloma/Lymphoma	46	20	12
Other	120	43	27
Total	607	176	98

**^a^** Motor status was unknown for 22 patients

**^b^** Pearson chi-square 2-sided test.

In 46 of 58 operated patients, the surgery was followed by RT, while 12 patients did not receive postoperative RT either because of complications associated with surgery or because of early death. 11 of 845 patients who started RT as primary treatment were later operated because of worsening of symptoms, 3 before and 8 after RT had been completed.

### Radiotherapy

8.0 Gy was used as single-fraction (SF) primary treatment in 141 patients and multiple- fraction (MF) treatment was used in 704 patients. In 1 of 4 RT centers, SF RT was used more frequently. The most frequently used MF schedules were 3.0 Gy × 10 in 554 patients, 4.0 Gy × 5 in 33 patients, and 3.0 Gy × 12 in 13 patients. 94% of the patients completed RT as initially scheduled. 73 patients were non-ambulatory (Frankel A–C) before the start of RT, 154 were ambulatory with minor motor deficit (Frankel D), and 596 patients had no motor impairment (Frankel E).

In the multiple logistic regression model, the type of primary tumor, age, and motor impairment were associated with the use of MF RT as opposed to SF RT (Table 2).

**Table 2.** Comparison of the use of multiple-fraction (MF) and single-fraction (SF) radiotherapy (RT) as primary treatment for spinal metastatic disease in 845 patients **^a^**

	n	MF	SF	p -value	OR (95% CI)
Primary cancer diagnosis				< 0.001	
Myeloma/lymphoma	60	56	4		1
Breast	149	135	14	0.7	0.8 (0.3–3.0)
Prostate	249	200	49	0.02	0.2 (0.1–0.8)
Lung	172	128	44	0.002	0.2 (0.1–0.5)
Kidney	37	34	3	0.5	0.5 (0.1–2.8)
Other	178	151	27	0.1	0.3 (0.1–1.1)
Age				0.04	
70+	407	323	84		1
50–69	375	325	50	0.01	1.8 (1.1–2.8)
19–49	63	56	7	0.4	1.4 (0.6–3.5)
Sex				0.5	
Female	345	294	51		
Male	500	410	90		
Motor impairment **^b^**				< 0.001	
Non-ambulatory (Frankel A–C)	73	69	4		1
Ambulatory with minor motor deficit (Frankel D)	154	141	13	0.4	0.6 (0.2–1.9)
Normal motor status (Frankel E)	596	486	110	0.005	0.2 (0.1–0.6)
Multiplicity of spine metastases				0.6	
One vertebra affected	116	99	17		
Multiple vertebra	729	605	124		
RT center				< 0.001	
Center 1	90	47	43		1
Center 2	429	385	44	< 0.001	8.3 (4.7–14.8)
Center 3	204	169	35	< 0.001	5.0 (2.7–9.2)
Center 4	122	103	19	< 0.001	5.4 (2.7–10.9)

OR: odds ratio for choice of MF RT vs. SF RT; 95%CI: 95% confidence interval.

**^a^** Binary logistic regression model.

**^b^** Motor impairment was unknown for 22 patients.

### Surgery

Motor impairment due to epidural compression was the indication for surgical treatment in 46 of 58 primarily operated patients, pain was the indication in 8 patients, 3 patients were operated due to spinal stenosis caused by tumor mass (but without motor impairment), and in 1 patient the need for biopsy was recorded as the indication for surgery. Posterior decompression and fixation with pedicle screws and rods was the most common technique (45 patients); 8 patients were operated with posterior decompression without fixation. 5 patients, all of them with metastatic lesions in the cervical spine, were operated with anterior approach and corporectomy. The reconstruction after corporectomy was done with cage in 2 patients and with autolog bone graft in 3 patients. 5 patients were reoperated due to deep infection or mechanical problems with fixation fixation of implants within 2 months.

### Use of primary surgery vs. radiotherapy

Surgical treatment was given to 25 of 98 non-ambulatory patients, to 22 of 176 patients with minor motor deficit (Frankel D), and to 11 of 607 patients without motor impairment (Frankel E) (Table 3). Consequently, 47 of 274 (17%) first-time admitted patients with any grade of motor compromise (Frankel A–D) underwent surgery as primary treatment. Primary cancer diagnosis, grade of motor impairment, male sex, and multiplicity of metastases in the spine were associated with the use of surgery as opposed to RT in the multiple binary logistic regression analyses (Table 3). Patients younger than 50 years appeared to be operated more often, but the difference was not statistically significant. The lowest proportion of patients who received primary surgery as opposed to RT was in the group of patients with breast cancer (2%), while 19 of 79 patients with myeloma or lymphoma were operated. Surgery was performed as primary treatment in 18% of the patients with solitary spine metastasis and only in 4% of the patients with multiple spine metastases (Table 3). There were no statistically significant differences in the distribution of operated patients between surgical centers regarding diagnoses or indications for surgery.

**Table 3.** Comparison of the use of surgery and radiotherapy (RT) as primary treatment of spinal metastatic disease in 903 patients **^a^**

	n	Primary surgery	Primary RT	p-value	OR (95% CI)
Primary cancer diagnosis				0.006	
Myeloma and Lymphoma	79	19	60		1
Breast	152	3	149	0.1	0.3 (0.1–1.5)
Prostate	260	11	249	0.008	0.2 (0.1–0.7)
Lung	179	7	172	< 0.001	0.1 (0.04–0.7)
Kidney	40	3	37	0.1	0.3 (0.07–1.4)
Other	193	15	178	0.003	0.2 (0.1–0.6)
Age				0.4	
70+	433	26	40		1
50–69	400	25	375	0.2	1.6 (0.8–3.2)
19–49	70	7	63	0.4	1.7 (0.5–6.0)
Gender				0.03	
Female	361	16	345		1
Male	542	42	500	0.03	2.5 (1.1–5.7)
Time from primary cancer diagnosis to treatment for SM ^**b**^				0.6	
Within one year	396	40	356		1
Between one and 5 years	279	11	268	0.2	0.6 (0.3–1.3)
6 years or more	204	5	199	0.08	0.4 (0.1–1.1)
Motor impairment ^**c**^				< 0.001	
Normal motor status (Frankel E)	607	11	596		1
Ambulating with minor motor deficit (Frankel D)	176	22	154	< 0.001	9 (4–21)
Non-ambulatory (Frankel A–C)	98	25	73	< 0.001	21 (9–50)
Multiplicity of spine metastases				< 0.001	
One vertebra affected	142	26	116		1
Multiple vertebra	761	32	729	< 0.001	0.2 (0.1–0.4)

OR: odds ratio for choice of surgery vs. RT; 95% CI: 95% confidence interval.

**^a^** Multiple binary logistic regression model.

**^b^** Time from primary cancer diagnosis to treatment for SM was unknown for 24 patients.

**^c^** Motor impairment was unknown for 22 patients.

### Survival

**Figure 1.**
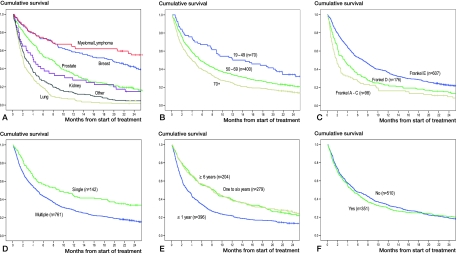
Kaplan-Meier plots with results of log-rank tests of survival related to pretreatment factors. A. Primary tumor (p < 0.001). B. Age (p < 0.001). C. Motor impairment ^a^ (p < 0.001). D. Multiplicity of metastases in spine (p < 0.001). E. Time from diagnosis of cancer to treatment ^b^ (p < 0.001). F. Metastases in spine at the time of primary cancer diagnosis ^c^ (p = 0.6).

The median survival after the first admission for treatment for SM was 5 (0–48) months (Table 4). 255 of 903 included patients (28%) died within 2 months after start of treatment. Survival after 1 year was 31%. Survival depended on primary tumor type, motor impairment prior to treatment, multiplicity of metastatic lesions in the spine, time from primary cancer diagnosis to admission for SM, and age. Presence of spine metastases at the time of cancer diagnosis was not a statistically significant predictive factor for survival (Figure 1).

**Table 4.** Median survival from start of treatment according to the primary cancer diagnosis, motor impairment, multiplicity of metastases in the spine, and age

	n	Median survival, months	95% CI
Primary tumor			
Myeloma/Lymphoma **^a^**	79	–	–
Breast	152	18.8	13.5–24.2
Prostate	260	7.6	6.0–9.2
Kidney	40	4.0	2.4–5.6
Other	193	2.7	2.0–3.4
Lung	179	2.0	1.7–2.4
Motor impairment ^**b**^			
Normal neurological status	607	7.0	5.9–8.2
Ambulatory with minor motor deficit (Frankel D)	176	3.4	2.2–4.6
Non-ambulatory	98	1.8	1.2–2.4
Multiplicity in spine			
Single	142	9.1	5.1–13.1
Not single	761	4.7	4.1–5.3
Age			
19–49	70	12.0	3.5–20.6
50–69	400	5.6	4.4–6.7
70+	433	4.0	3.2–4.8
All included patients	903	5.1	4.4–5.7

**^a^** 42 of 79 patients with myeloma/lymphoma were alive at the follow-up.

**^b^** Motor impairment was unknown for 22 patients.

70 of 79 patients with myeloma or lymphoma, 134 of 152 patients with breast cancer, and 211 of 260 patients with prostate cancer were alive 2 months after treatment. The lowest 2-month survival was in patients with melanoma (15 of 31 patients still alive), lung cancer (93 of 179), unknown primary site (25 of 45), gastrointestinal cancer (42 of 73), and kidney cancer (26 of 40 patients still alive).

487 of 607 patients (80%) with normal neurological status, 113 of 176 ambulatory patients (64%) with minor neurological compromise (Frankel D), and 45 of 98 non-ambulatory patients were alive after 2 months. 84% (119 of 142) of the patients with a single lesion in the spine and 70% (529 of 761) with multiple lesions survived more than 2 months.

**Figure 2.**
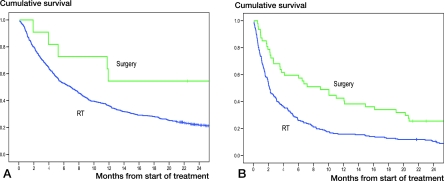
Kaplan-Meier plots with results of log-rank test of overall survival after surgery and radiotherapy (RT) for patients without motor impairment (Frankel E) (panel A; p = 0.03), and for patients with motor impairment (Frankel A–D) (panel B; p < 0.001).

11 of 58 patients who underwent surgery as primary treatment, 185 of 704 of those who received MF RT, and 59 of 141 of those who received SF RT died within 2 months after the start of treatment. Overall survival was better in the surgery group—in the patients both with and without motor impairment (Figure 2).

196 of 255 patients (77%) who died within 2 months after the start of treatment underwent either surgery or MF RT, and 84 of those patients had normal neurological status.

## Discussion

Our data on the use of surgery and RT for SM are based on a geographically defined patient population, which reduces the impact of selection bias in the results presented. The indications for RT are complex, as RT may be used to target pain control in patients with uncomplicated painful vertebral metastases and in patients with poor performance status and short life expectancy, even in the presence of a motor deficit. However, we assume that both local tumor control and pain were the indications for RT in the majority of patients who received RT.

Clinically significant neural compression and spinal instability are the most common indications for surgical treatment, together with non-response to prior RT (Harrington 1997, Tomita et al. 2001). Patients with short life expectancy should be identified to avoid time-consuming treatment requiring long hospital stay, such as surgery or MF RT. Our study showed that a high percentage of patients who received such time-consuming treatment died within 2 months. This may be partly explained by clinicians' inability to reliably identify patients who are at considerable risk of dying within just a few weeks. Estimation of expected survival is usually based on doctors' clinical experience. However, different scoring systems predicting survival for patients with SM have been elaborated in order to identify a “no surgery” group (Bauer and Wedin 1995, Enkaoua et al. 1997, Tomita et al. 2001, van der Linden et al. 2005, Tokuhashi et al. 2005, Leithner et al. 2008, White et al. 2008). In these systems, the lowest expected survival groups are usually associated with 3–6 months of expected survival time. However, the problem is rather to identify the patients who are likely to die from cancer within a few weeks—in order to avoid over-treatment, which would make their last weeks unnecessarily difficult (Bauer 2005). Motor impairment, especially inability to walk, has been identified as a factor that is predictive of poor survival and—together with performance status—has been used in different survival scores (Leithner et al. 2008). Motor impairment is, however, difficult to use in the selection of patients for “no surgery”, as this parameter is a main indication for surgical treatment.

Lung cancer as a primary diagnosis has been recognized as a prognostic factor for poor survival in both our study and in several other studies. However, we found that there was no substantial difference in the percentage of primarily operated patients with lung cancer and patients with diagnoses associated with better expected survival, such as breast or prostate cancer. This shows that the primary cancer diagnosis as a risk factor for poor survival was not always taken in account when selecting patients for surgical treatment.

Myeloma and lymphoma are radiosensitive tumors; however, a high percentage of patients with these diagnoses were operated on. This can be explained by the higher percentage of patients with motor impairment in this group, and by better survival expectancy. Another explanation might be a more rapid development of motor deficits in these patients. Furthermore, bony metastatic lesions from lymphoma and myeloma are usually osteolytic, which may give mechanical instability and indication for surgery, especially in the case of pathological fractures and medulla compression by bony fragments. In such cases, RT is often considered less effective than surgery. We found no statistically significant differences in the distribution of operated patients between surgical centers regarding diagnoses or indications for surgery, but different traditions in selecting patients for surgery in different centers could cause some bias.

The low percentage of operated patients with breast and prostate cancer may be explained by low percentages of non-ambulatory patients, especially in patients with breast cancer. This can be explained by slow growth of the tumors, but also by a traditionally better follow-up and early referral for treatment of patients with breast cancer. Furthermore, in addition to RT, other non-invasive treatment options such as hormone therapy and chemotherapy are efficient treatment options in these patients.

Although male sex was associated with more frequent use of surgery in the multiple model, this finding should be interpreted with caution because of the clear connection between patient sex and some cancer diagnoses.

The 3-month survival following surgical treatment that we found (72%) is similar to the outcome presented in a population-based study from Canada (Finkelstein et al. 2003) (71%). In a single-institution study from Sweden (Jansson and Bauer 2006), a postoperative 90-day survival of 63% was found. We assume that the choice of treatment modality within the palliative framework does not affect survival in each particular case. The higher survival in the surgery group that we found most likely reflects selection of patients with a better prognosis.

RT was used as primary treatment for the majority of our patients, and most patients had MF RT rather than SF RT. There is still no consensus about the most appropriate RT schedule for SM. Rades et al. (2005) and Kaasa et al. (2006) recommended 1 × 8 Gy for patients with poor predicted survival and for pain treatment, and 3 Gy × 10 for other patients. In our series, most patients (82%) received MF RT. We expected that patients with short survival would predominate in the SF group, but even among those who died within 2 months, this regime was used in only one third of the cases. The treatment time with SF is 1 day, and is preferable for patients with a short life expectancy. Better clinical guidelines in the selection of patients for RT regimens are needed.

Was the balance between the use of RT or surgery in our study population adequate according to the accepted guidelines? Many patients with motor compromise have advanced disease, poor performance status, multiple levels of cord compression, and short survival, and—consequently—are not candidates for surgical treatment. Half of our patients with neurological impairment who were not operated, but underwent RT, died within 2 months. We believe that only patients with motor impairment and more than 2 months of expected survival time should be considered for surgical treatment. In our study, 121 patients (13% of all patients) with motor impairment underwent RT and survived more than 2 months. Some of the 121 irradiated patients with motor compromise may have benefited from operation. However, we assume that the ratio between RT and surgery in patients with motor impairment was reasonable.

Only 2% of the patients with Frankel E were operated, and possibly more patients in this group could have benefited from surgery. Pain as an indication for surgery in patients with SM is, however, controversial. RT has been an established treatment for metastatic bone pain for decades. Prospective studies comparing pain relief as outcome after RT and surgery are needed.

The limitation of our study is the retrospective design, which made it impossible to obtain reliable data about motor function at follow-up. Information on factors that have previously been shown to be of prognostic value for post-treatment survival, such as performance status and presence of visceral metastases (van der Linden et al. 2005, Leithner et al. 2008), was not available in this study.

## References

[CIT0001] Abrahm JL, Banffy MB, Harris MB (2008). Spinal cord compression in patients with advanced metastatic cancer: ”all I care about is walking and living my life”. JAMA.

[CIT0002] Bauer HC (2005). Controversies in the surgical management of skeletal metastases. J Bone Joint Surg (Br).

[CIT0003] Bauer HC, Wedin R (1995). Survival after surgery for spinal and extremity metastases. Prognostication in 241 patients. Acta Orthop Scand.

[CIT0004] Bruland O, Hird A, Chow E, Compston J.E., Lian J.B (2009). Radiotherapy of Skeletal Metastases. Primer on the Metabolic Bone Diseases and Disorders of Mineral Metabolism.

[CIT0005] Chow E, Harris K, Fan G, Tsao M, Sze WM (2007). Palliative radiotherapy trials for bone metastases: a systematic review. J Clin Oncol.

[CIT0006] Conway R, Graham J, Kidd J, Levack P (2007). What happens to people after malignant cord compression? Survival, function, quality of life, emotional well-being and place of care 1 month after diagnosis. Clin Oncol (R Coll Radiol).

[CIT0007] Enkaoua EA, Doursounian L, Chatellier G, Mabesoone F, Aimard T, Saillant G (1997). Vertebral metastases: a critical appreciation of the preoperative prognostic tokuhashi score in a series of 71 cases. Spine.

[CIT0008] Finkelstein JA, Zaveri G, Wai E, Vidmar M, Kreder H, Chow E (2003). A population-based study of surgery for spinal metastases. Survival rates and complications. J Bone Joint Surg (Br).

[CIT0009] Frankel HL, Hancock DO, Hyslop G, Melzak J, Michaelis LS, Ungar GH, Vernon JD, Walsh JJ (1969). The value of postural reduction in the initial management of closed injuries of the spine with paraplegia and tetraplegia. I. Paraplegia.

[CIT0010] Harrington KD (1997). Orthopedic surgical management of skeletal complications of malignancy. Cancer.

[CIT0011] Hartsell WF, Scott CB, Bruner DW, Scarantino CW, Ivker RA, Roach M, Suh JH, Demas WF, Movsas B, Petersen IA, Konski AA, Cleeland CS, Janjan NA, DeSilvio M (2005). Randomized trial of short- versus long-course radiotherapy for palliation of painful bone metastases. J Natl Cancer Inst.

[CIT0012] Jansson KA, Bauer HC (2006). Survival, complications and outcome in 282 patients operated for neurological deficit due to thoracic or lumbar spinal metastases. Eur Spine J.

[CIT0013] Kaasa S, Brenne E, Lund JA, Fayers P, Falkmer U, Holmberg M, Lagerlund M, Bruland O (2006). Prospective randomised multicenter trial on single fraction radiotherapy (8 Gy x 1) versus multiple fractions (3 Gy x 10) in the treatment of painful bone metastases. Radiother Oncol.

[CIT0014] Klimo P, Thompson CJ, Kestle JR, Schmidt MH (2005). A meta-analysis of surgery versus conventional radiotherapy for the treatment of metastatic spinal epidural disease. Neuro Oncol.

[CIT0015] Leithner A, Radl R, Gruber G, Hochegger M, Leithner K, Welkerling H, Rehak P, Windhager R (2008). Predictive value of seven preoperative prognostic scoring systems for spinal metastases. Eur Spine J.

[CIT0016] Loblaw DA, Laperriere NJ, Mackillop WJ (2003). A population-based study of malignant spinal cord compression in Ontario. Clin Oncol (R Coll Radiol).

[CIT0017] Loblaw DA, Perry J, Chambers A, Laperriere NJ (2005). Systematic review of the diagnosis and management of malignant extradural spinal cord compression: the Cancer Care Ontario Practice Guidelines Initiative's Neuro-Oncology Disease Site Group. J Clin Oncol.

[CIT0018] Patchell RA, Tibbs PA, Regine WF, Payne R, Saris S, Kryscio RJ, Mohiuddin M, Young B (2005). Direct decompressive surgical resection in the treatment of spinal cord compression caused by metastatic cancer: a randomised trial. Lancet.

[CIT0019] Prasad D, Schiff D (2005). Malignant spinal-cord compression. Lancet Oncol.

[CIT0020] Rades D, Stalpers LJ, Veninga T, Schulte R, Hoskin PJ, Obralic N, Bajrovic A, Rudat V, Schwarz R, Hulshof MC, Poortmans P, Schild SE (2005). Evaluation of five radiation schedules and prognostic factors for metastatic spinal cord compression. J Clin Oncol.

[CIT0021] Rades D, Fehlauer F, Schulte R, Veninga T, Stalpers LJ, Basic H, Bajrovic A, Hoskin PJ, Tribius S, Wildfang I, Rudat V, Engenhart-Cabilic R, Karstens JH, Alberti W, Dunst J, Schild SE (2006). Prognostic factors for local control and survival after radiotherapy of metastatic spinal cord compression. J Clin Oncol.

[CIT0022] Sande TA, Ruenes R, Lund JA, Bruland OS, Hornslien K, Bremnes R, Kaasa S (2009). Long-term follow-up of cancer patients receiving radiotherapy for bone metastases: results from a randomised multicentre trial. Radiother Oncol.

[CIT0023] Tokuhashi Y, Matsuzaki H, Oda H, Oshima M, Ryu J (2005). A revised scoring system for preoperative evaluation of metastatic spine tumor prognosis. Spine.

[CIT0024] Tomita K, Kawahara N, Kobayashi T, Yoshida A, Murakami H, Akamaru T (2001). Surgical strategy for spinal metastases. Spine.

[CIT0025] van der Linden YM, Lok JJ, Steenland E, Martijn H, van HH, Marijnen CA, Leer JW (2004). Single fraction radiotherapy is efficacious: a further analysis of the Dutch Bone Metastasis Study controlling for the influence of retreatment. Int J Radiat Oncol Biol Phys.

[CIT0026] van der Linden YM, Dijkstra SP, Vonk EJ, Marijnen CA, Leer JW (2005). Prediction of survival in patients with metastases in the spinal column: results based on a randomized trial of radiotherapy. Cancer.

[CIT0027] White BD, Stirling AJ, Paterson E, squith-Coe K, Melder A (2008). Diagnosis and management of patients at risk of or with metastatic spinal cord compression: summary of NICE guidance. BMJ.

[CIT0028] Young RF, Post EM, King GA (1980). Treatment of spinal epidural metastases. Randomized prospective comparison of laminectomy and radiotherapy. J Neurosurg.

